# Vascular-type Ehlers-Danlos syndrome caused by a hitherto unknown genetic mutation: a case report

**DOI:** 10.1186/1752-1947-7-35

**Published:** 2013-02-01

**Authors:** Fumihiro Kashizaki, Atsushi Hatamochi, Kazunori Kamiya, Akira Yoshizu, Hiroaki Okamoto

**Affiliations:** 1 Department of Respiratory Medicine, Yokohama Municipal Citizen’s Hospital, 56 Okazawa-cho, Hodogaya-ku, Yokohama, Kanagawa, 240-8555, Japan; 2 Department of Medical Oncology, Yokohama Municipal Citizen’s Hospital, 56 Okazawa-cho, Hodogaya-ku, Yokohama, Kanagawa, 240-8555, Japan; 3 Department of Dermatology, School of Medicine, Dokkyo Medical University, 880 Kitakobayashi, Mibu, Tochigi, 321-0293, Japan; 4 Department of Thoracic Surgery, Yokohama Municipal Citizen’s Hospital, 56 Okazawa-cho, Hodogaya-ku, Yokohama, Kanagawa, 240-8555, Japan

## Abstract

**Introduction:**

Vascular-type Ehlers-Danlos syndrome is an autosomal dominant disease that causes arterial spurting, intestinal perforation, uterine rupture and hemopneumothorax due to decreased production of type III collagen. The average age at death is 48 years old, and it is considered to be the most severe form of Ehlers-Danlos syndrome. We report the case of a 64-year-old Japanese woman and her 38-year-old daughter who were diagnosed with this disease.

**Case presentation:**

A 64-year-old Japanese woman was referred to our hospital because of right anterior chest pain following cough and pharyngeal discomfort. Pleurisy was suspected due to the presence of right pleural effusion, so the next day she was referred to our department, where a detailed examination led to the diagnosis of hemothorax. The bleeding that caused the right hemothorax was difficult to control, so our patient was transferred to the Department of Thoracic Surgery for hemostasis control. Our patient’s personal history of uterine hemorrhage and skin ulcers, as well as the finding of skin fragility during surgery, were indicative of a weak connective tissue disease; therefore, after improvement of the hemothorax, a genetic analysis was performed. This revealed a heterozygous missense mutation in *COL3A1*, c.2411 G>T p.Gly804Val (exon 36). A detailed investigation conducted at a later date revealed that her daughter also had the same genetic mutation. This led to the diagnosis of vascular-type Ehlers-Danlos syndrome characterized by a new gene mutation.

**Conclusion:**

We report a new genetic mutation associated with vascular-type Ehlers-Danlos syndrome. We present the clinical and imaging findings, and the disease and treatment course in this patient. We believe this information will be important in treating future cases of vascular-type Ehlers-Danlos syndrome in patients with this mutation.

## Introduction

Ehlers-Danlos syndrome (EDS) is used to refer to a group of genetic disorders characterized by connective tissue fragility caused by abnormalities affecting collagen - a molecular constituent of the extracellular matrix - as well as its modifying enzymes [1]. EDS is classified into six different types: classical, joint hypermobility, vascular, kyphoscoliosis, arthrochalasia and dermatosparaxis [1]. The vascular type (vEDS), which is considered to have the most unfavorable prognosis, is found in one person per 50,000 to 100,000. vEDS is caused by mutations in the alpha 1 type III collagen gene (*COL3A1*), which result in decreased production of type III collagen [2]. Although it is believed to be an autosomal dominant disease, half of the affected patients reportedly have no family history, and it is assumed that they develop the disease through newly generated mutations [3]. So far, over 250 cases of mutations of the *COL3A1* gene have been reported, and no particular site seems to have a high proportion of mutations [4]. In addition, the relationship between the site of mutation and the severity of the disease still remains unclear [4]. The diagnosis of vEDS is based on clinical findings and confirmed by identification of a causative mutation in *COL3A1*[1].

In this study, we report our experience with a case of vEDS in which the diagnosis was based on clinical findings and confirmed by genetic analysis, which showed a heterozygous missense mutation, c.2411 G>T p.Gly804Val (exon 36), of the *COL3A1* gene.

## Case presentation

A 64-year-old Japanese woman experienced pharyngeal discomfort late one night in July 2011, as well as pain in the right anterior region of her chest while coughing. She was brought to our hospital on the same night because the chest pain continued. A detailed examination showed right pleural effusion, based on which pleurisy was diagnosed. She was hospitalized on the day of admission and referred to our department the next day. Her past medical history revealed that, at the age of 26 years, she gave birth to her first child at 36 weeks of pregnancy, and underwent a total hysterectomy one month later because of an intractable uterine hemorrhage. At the age of 49, she underwent stripping of varicose veins of her lower extremities, and at the age of 62 years, she was diagnosed with arteriovenous fistulae of her lower extremities, for which she was being treated at a nearby dermatology clinic on an outpatient basis. She had a medical history of a skin ulcer, but had no history of easy and extensive bruising of the skin, enlargements of scars, or postoperative complications. There was no family history of vEDS, and our patient did not have a history of smoking or alcohol consumption.

**Figure 1:**
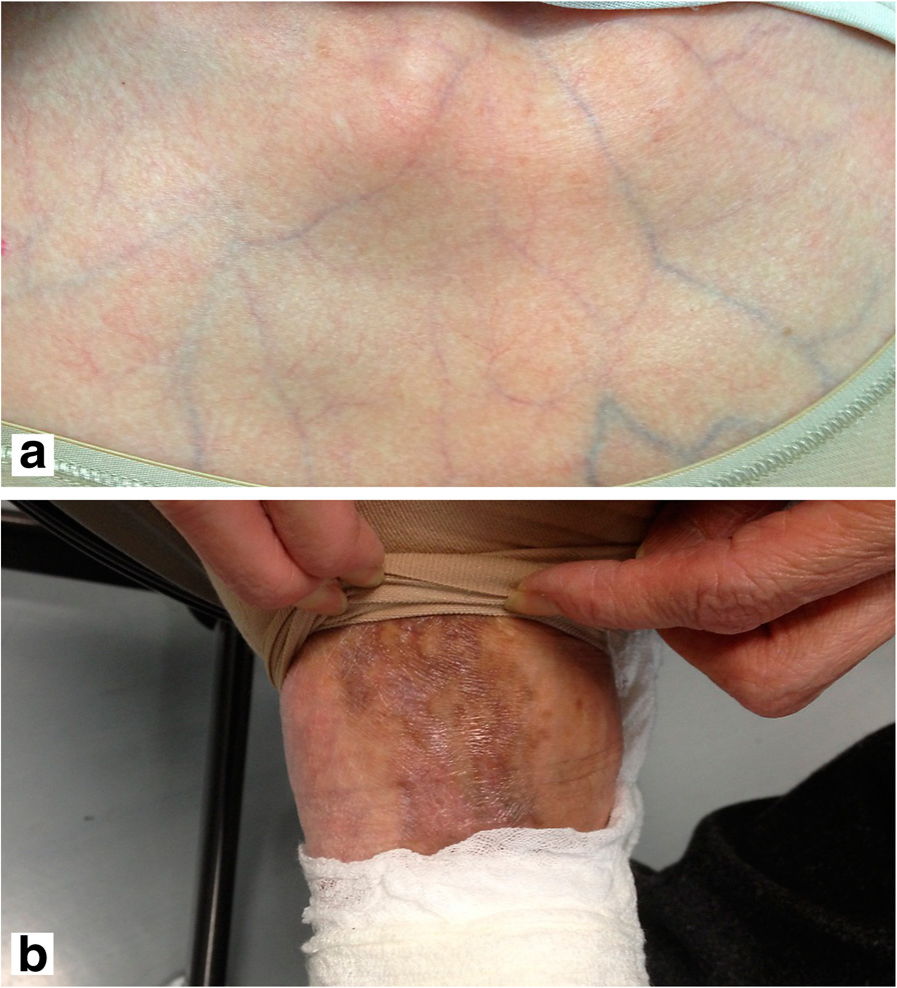
**Skin findings. (a)** Anterior chest wall: the skin was thin and subcutaneous veins were visible through it. **(b)** Back of the left foot: pigmented scars caused by cutaneous arteriovenous fistulae were visible.

The physical findings on admission were as follows: body temperature, 35.7°C; blood pressure, 94/60mmHg; pulse rate, regular at 67 beats/min; respiratory rate, 16 beats/min. Percutaneous monitoring of her arterial oxygen saturation in room air showed a blood oxygen saturation (SpO2) level of 95%. Our patient was conscious and lucid, and there were no abnormal findings on her neck. There was no abnormal heart sound, but her breathing sounds were diminished on the right side. The site of the right chest pain was around the second intercostal space at the right sternal border, and no tenderness was observed. There were no significant abdominal or neurological findings. Her face was normal. Thin subcutaneous veins were visible through the skin of the anterior chest region (Figure 1a), and pigmented scars due to cutaneous arteriovenous fistulae were found at the dorsum of her left foot (Figure 1b). Premature aging of her skin was found.

**Figure 2:**
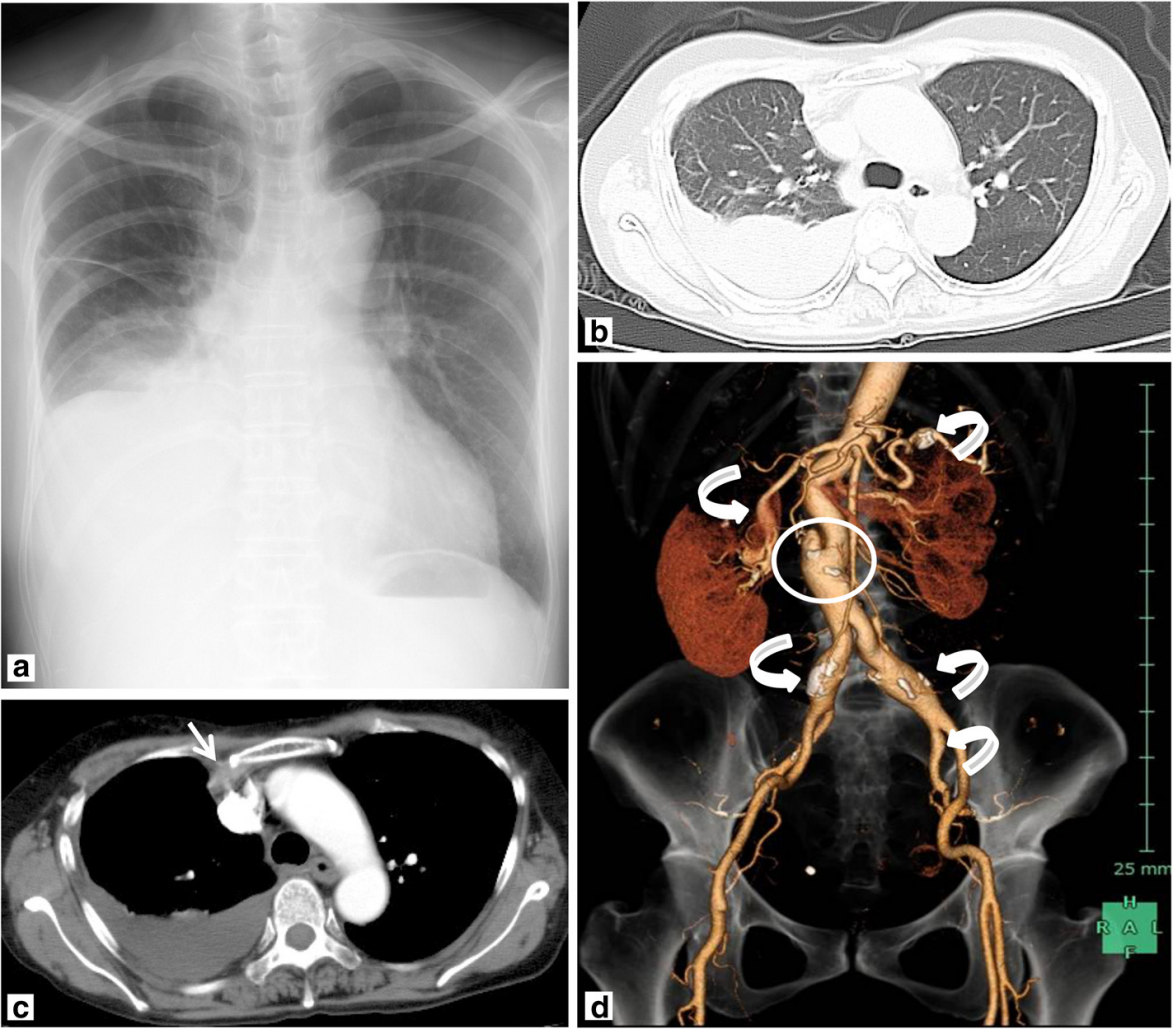
**Chest and abdominal imaging findings. (a)** Plain chest radiograph: pleural effusion was found in the right hemithorax. **(b)** Chest computed tomography: right pleural effusions were detected in the lung window. **(c)** In the mediastinal window, the right pleural effusion was 30 to 60 Hounsfield units; a low-density area (white arrow) was found around the right internal thoracic artery. **(d)** Abdominal computed tomography: saccular/fusiform aneurysms (white curved arrows) were found in the celiac artery, splenic artery, superior mesenteric artery, renal arteries and common iliac artery. Aortic dissection was found (white circle).

On admission, the collected blood samples showed an erythrocyte sedimentation rate of 56mm/h and immunoglobulin G levels that had elevated to 2025mg/dL. Her activated partial thromboplastin time, prothrombin time, fibrinogen level, D-dimer level and other laboratory findings were normal (Table 1). A plain chest radiograph showed a right pleural effusion (Figure 2a); chest computed tomography (CT) showed pleural fluid with a CT value of 30 to 60 Hounsfield units in the right pleural cavity, which was accompanied by a region showing CT values comparable to those of pleural effusion, located dorsally from the area surrounding her right internal thoracic artery (Figure 2b,c). An abdominal CT showed a large number of saccular and fusiform aneurysms in her celiac artery, splenic artery, superior mesenteric artery, right and left renal arteries, and right and left common iliac arteries (Figure 2d).

**Table 1:** Laboratory findings on admission

**Hematology**	**Reference range**	**On admission this hospital**	**Coagulation**	**Reference range**	**On admission this hospital**
White-cell count	3000-9000	6400/mm^3^	Activated partial thromboplastin time	28-38	29.4 seconds
Neutrophils	41-79	46%	Thromboplastin time and international normalized ratio	0.9-1.0	0.98
Lymphocytes	21-51	44%			
Eosinophils	0.3-6.0	2%			
Hemoglobin	11.0-15.0	11.1g/dL	Fibrinogen	130-380	318mg/dL
Platelet	14.0-35.0 × 10^4^/mm^3^	13.6 × 10^4^/mm^3^	D-dimer	0.0-1.0	1.0μg/mL
Erythrocyte sedimentation rate	2-16	56mm/hr	**Sputum**		
			bacterial (culture)	normal flora	normal flora
			acid-fast bacilli		
**Biochemistry**			smear	negative	negative
Lactate dehydrogenase	110-210	202IU/L	culture	negative	negative
Creatine kinase	45-170	101IU/L	**Pleural effusion**		
			Cytology		class II
**Serological test**					
C-reactive protein	0.0-0.3	0.2mg/dL	**Urinalysis**		
Immuno-globulin G(IgG)	870-1700	2025mg/dL	protein	( - )	( - )
IgG4	4.8-105	48mg/dL	blood	( - )	( - )
Carcino-embryonic antigen	0.0-5.0	0.8ng/mL			
Myeloperoxidase-type anti-neutrophil cytoplasmic antibody	<10	<10U/mL			

Blood samples collected eight hours after admission, at the time of referral to our department, showed hemoglobin levels of 9.5g/dL, and a plain chest radiograph showed an increase in the right pleural effusion compared with the image taken at the time of admission. Therefore, an exploratory pleural puncture was performed and a bloody pleural effusion was obtained. A drain was inserted into her thoracic cavity. Approximately 600mL of bloody pleural effusion was recovered during the two hours that followed the insertion of the drain. Her lung expansion was poor, so emergent irrigation and drainage and hemostatic surgery were performed using thoracoscopy. Surgical findings revealed the presence of an ecchymosis behind the mediastinal pleura that extended from the posterior surface of her sternum towards her right atrium. However, the site of bleeding, adhesions and cysts that caused the hemothorax were not found in her lung parenchyma or chest wall. Because the connective tissue was fragile, and the surgery could no longer be postponed, we affixed fibrin glue to the ecchymosis and completed the surgical procedure. Postoperative reaccumulation of the pleural effusion was not observed, and a blood transfusion was not required.

**Figure 3:**
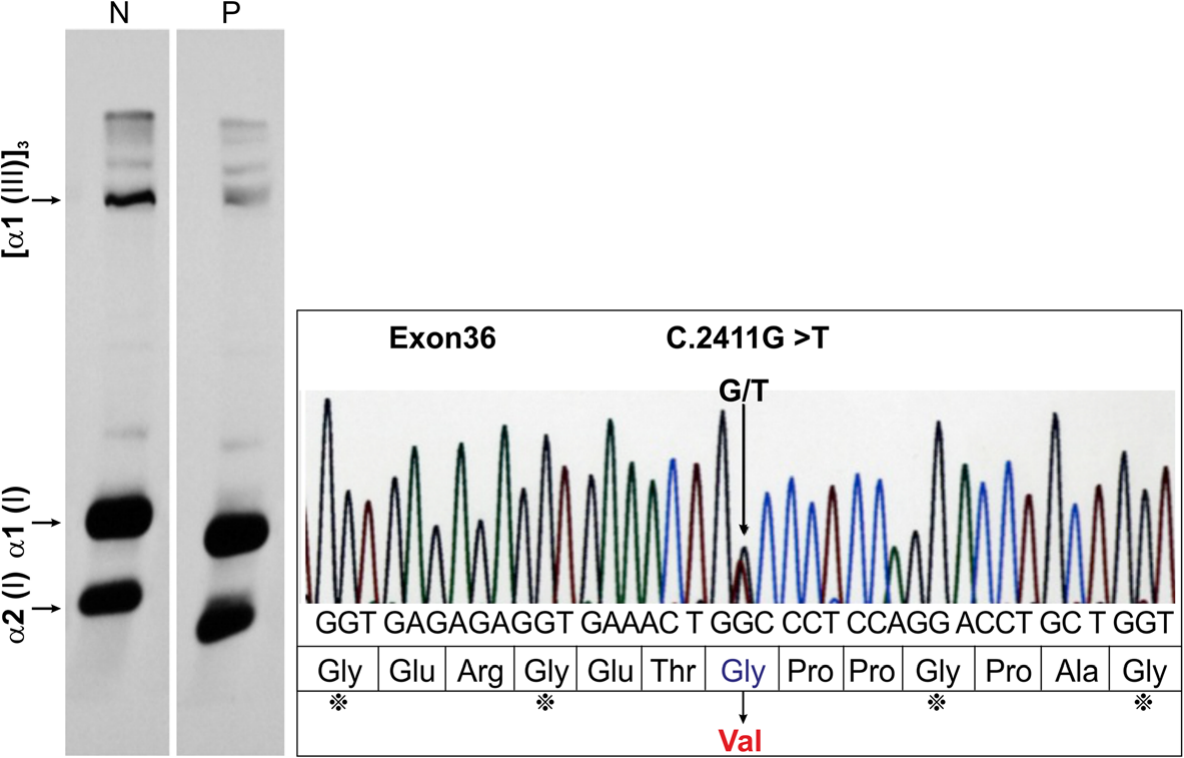
**Type III collagen production capacity and analysis of the *COL3A1 *gene. (a)** Type III collagen production capacity: the patient (P) showed decreased expression of [α1(III)] 3 compared with the normal control (N). The production of type III/type I collagen was 14.7% of the normal value. **(b)** Analysis of the *COL3A1* gene: a heterozygous missense mutation characterized by GGC(Gly)→GTC(Val) was found at c. 2411 (c.2411 G>T p.Gly804Val (exon 36)). α1(I), α1 chain of type I collagen; α2(I), α2 chain of type I collagen; Gly, glycine; Glu, glutamic acid; Arg, arginine; Thr, threonine; Pro, proline; Ala, alanine.

These skin findings, her past medical history and the presence of a large number of aneurysms were indicative of weak connective tissue disease. Our patient met two major diagnostic criteria for vEDS according to Beighton *et al*. [1], thin translucent skin and arterial, intestinal or uterine fragility or rupture. For a definite diagnosis of vEDS, a biopsy examination of skin from her upper right arm was performed, along with analysis of the type III collagen production capacity of cultured dermal fibroblasts and an analysis of the *COL3A1* gene. Histopathological examination of skin tissue sections stained with hematoxylin-eosin showed normal results. An electron microscope examination revealed that the collagen fibers were of an irregular size, which was most marked in regions surrounding the blood vessels. The amount of type III/I collagen had decreased by 14.7%, and a heterozygous missense mutation, c.2411 G>T p.Gly804Val (exon 36), was found in the *COL3A1* gene. These findings confirmed the diagnosis of vEDS (Figure 3).

Approximately one month after surgery for the hemothorax, our patient was transported to our emergency department because of sudden abdominal pain. The clinical and surgical findings led to the diagnosis of acute peritonitis due to perforation of her digestive tract. Our patient was discharged from the hospital one month after laparotomy and drainage. In addition, our patient had a 38-year-old daughter, who was single. The daughter was asymptomatic, but we conducted the genetic tests because vEDS is an autosomal dominant inherited disease. The results showed that she carried the same genetic mutation as her mother. Currently, she and her mother are being treated on an outpatient basis.

## Discussion

Approximately 98% of vEDS cases are caused by a mutation in the *COL3A1* gene. *COL3A1* is composed of 52 exons, and is expressed in the arteries, intestines, lungs and the uterus [5]. Mutations of this gene cause aortic aneurysms, aortic dissection and rupture, gastrointestinal hemorrhage and perforation, uterine rupture, pneumothorax, hemothorax, and, in some cases, sudden death [4]. In most cases, because the glycine that is present in the *COL3A1* gene is substituted for other amino acids, the generation of the normal type III collagen is inhibited, and vEDS develops [6]. In our study, our patients also showed a mutation in exon 36 of the *COL3A1* gene. This is the first time this heterozygous missense mutation, c.2411 G>T p.Gly804Val (exon 36), has been reported. Although this mutation, c.2411G>T p. G804V, is novel, mutation in the same gene of the same amino acid, c.2410G>A p.G804S, has been reported before [7].

In adults, symptoms of vEDS are mostly manifested in the cardiovascular system, respiratory system and digestive organs, where this gene is expressed, and are often observed during pregnancy and childbirth. Arterial rupture, arterial dissection and aneurysms are the cardiovascular symptoms reported in 77% of patients [8]. Arterial rupture may occur spontaneously in the thorax or abdomen in 50% of patients [2]. Intestinal rupture, mostly sigmoid, has been reported in 25% of patients with vEDS [2]. Hemopneumothorax, hemoptysis and alveolar hemorrhage have been found in 51% of patients, and have been reported to be the first symptom in 45% of patients [9]. Kawabata *et al*. previously reported that most pulmonary lesions consist of idiopathic lung tissue lacerations [10]. Chest CT has been reported to show ground glass opacity due to bleeding, a tumor-like shadow due to the presence of a hematoma, a cavernous opacity following the formation of the hematoma, or a cystic shadow and a calcified opacity [6,8,11,12]. In addition, most pleural lesions have been reported to consist of pneumothorax and hemothorax caused by weakening of the pleura and vascular fragility [2,6,11-13]. Our patient had gastrointestinal perforation and hemothorax, which are very similar to the complications associated with vEDS.

vEDS is associated with a maximal mortality risk of 12% due to uterine rupture before or after childbirth [2]. Thus, most symptoms of vEDS found in adulthood are severe; one quarter of patients will have experienced symptoms by the age of 20 years, and 80% of patients by the age of 40 years. The average age at the time of death has been reported to be 48 years [2,4]. In our case, the bleeding following childbirth at the age of 26 years is now believed to be caused by vEDS. Compared to the average age at the time of death reported thus far, our patient has a relatively long-term survival period, the reason for which is unknown. Pepin *et al*. reported that the complications of vEDS were not associated with specific mutations in *COL3A1*[4]. In our case, except for the fact that our patient was initially diagnosed at an advanced stage, there was no particularly unusual clinical phenotype.

No causal treatment for vEDS is available, and the current treatment consists mainly of the prevention of complications, as well as symptomatic treatment, including surgery and catheterization [2]. Because catheterization and surgical treatment are highly invasive [7], conservative treatment is preferred where possible, and when surgery is inevitable the procedure is performed cautiously. The postoperative monitoring needs to be prolonged. Any kind of elective surgery, for example, stripping of varicose veins, is contraindicated. Arteriograms and endoscopies should be avoided [14]. However, because we unfortunately did not notice the presence of vEDS at the time of admission, an emergent surgery for hemopneumothorax was performed, and our patient was consequently correctly diagnosed with vEDS based on the operative and pathological findings. At the time of the second admission, we believe that the emergent operation was absolutely necessary to rescue this patient from life-threatening acute peritonitis due to perforation of her digestive tract. Celiprolol has recently been reported to be effective in the prevention of arterial complications; it is a β1 receptor antagonist that is widely used in the treatment of hypertension. In a report on 53 patients with vEDS divided into a celiprolol administration group (25 patients) and a non-administration group (28 patients), the incidence of arterial ruptures caused by aortic dissection was 20% in the celiprolol group and 50% in the control group, and the hazard ratio was 0.36. Celiprolol was found to have a preventive effect on arterial rupture and aortic dissection that is brought about via an increase in type III collagen production via transforming growth factor-beta (TGFβ) stimulation or activation of intrinsic TGFβ [15]. However, the role of the TGFβ signal pathway in the treatment of vEDS using celiprolol is still debatable. Our patient was not given celiprolol, but her blood pressure was consistently maintained at a lower level. Thus, hemodynamic control may also be important for the treatment of vEDS.

In addition, because vEDS is an autosomal dominant inherited disorder, taking the next generation into consideration is necessary. To this end, psychosocial support and genetic counseling are of crucial importance. We conducted a genetic test for vEDS and found that the daughter carried the same genetic mutation as her mother. Because of this diagnosis, a systemic search for the lesion caused by vEDS and counseling by a specialized counselor were provided to the daughter.

## Conclusion

The case reported in this study is valuable because of the discovery of a new genetic mutation associated with vEDS. We have reported the clinical and imaging findings, and the disease and treatment course in our patient. We believe this data will be important in treating future cases of patients with vEDS with this mutation.

## Consent

Written informed consent was obtained from both patients for publication of this case report and accompanying images. A copy of the written consent is available for review by the Editor-in-Chief of this journal.

## Competing interests

The authors declare that they have no competing interests.

## Authors’ contributions

FK analyzed and interpreted the patient’s data and wrote the paper. AH conducted the genetic analysis. AH and HO were major contributors in writing the manuscript. KK and AY performed surgery. All authors read and approved the final manuscript.
